# Quantile Regression of Cognitive Ability and Achievement Inequality Before and After the Pandemic in One State

**DOI:** 10.3390/jintelligence14030036

**Published:** 2026-03-02

**Authors:** Al Mansor Helal, Jonathan Wai

**Affiliations:** 1 Department of Education Reform, University of Arkansas, Fayetteville, AR 72701, USA; jwai@uark.edu; 2 Department of Psychology, University of Arkansas, Fayetteville, AR 72701, USA

**Keywords:** quantile regression, pandemic learning loss, pandemic recovery, cognitive abilities, achievement vs. ability

## Abstract

This study draws from two cohorts of students (total N = 10,508) to examine cognitive ability and achievement inequality around the COVID-19 pandemic. Before and after the pandemic, two groups of Arkansas students were given both the Cognitive Abilities Test (CogAT), which is considered more of an ability or reasoning measure, and the ACT-Aspire, which is considered more of an achievement measure. We use a quantile regression framework to examine possible distributional impacts of the COVID-19 pandemic. Results demonstrate that the cognitive ability and/or achievement gaps did not widen after the pandemic, instead they stayed stable or narrowed moderately across groups. Results also indicate that cognitive ability was a significant and consistent predictor of achievement before COVID-19, but the strength of this relationship attenuated noticeably after the pandemic. This work helps add to the literature on COVID learning changes (large losses for many students), drawing from a large database of students in one state.

## 1. Introduction

The COVID-19 pandemic disrupted many aspects of global society, including education (Jakubowski et al., 2025), which led to what many education thought leaders and researchers are calling “learning losses” (e.g., Adelman, 2025; Cruz et al., 2025; Fahle et al., 2024). Other scholars have sought to place the pandemic education disruption in the context of history. For example, Hanushek (2025) points out that the decline in educational outcomes was occurring before the pandemic took place. Hanushek also argues that learning outcome decreases have genuine long-term impacts, even on GDP, arguing that by going back to examine historical disruptions in the past, we see that: “Traditional reform approaches have failed consistently. Despite decades of incremental changes, increased funding, and various educational reforms since the publication of A Nation at Risk in 1983, average performance has stagnated, and large disparities persist.” Within the U.S., different states have shown wide variation in their learning losses and recovery. For example, according to Fahle et al. (2024), after the pandemic, Alabama recovered well in math, and Louisiana, Illinois, and Mississippi recovered well in reading. However, in Arkansas, the state showed overall losses, ranking roughly in the middle of the pack compared to the rest of the nation. At the same time, specific districts such as Bentonville and Rogers (both in the region known as Northwest Arkansas) were doing relatively well (Stockwell, 2024).

Historically, at least in the wide-ranging fields that compose the education research and policy community, test scores are considered the developed level of achievement or human capital of groups of students at a particular time. There are some scholars who have pointed out that in high stakes situations test scores are often inflated (Koretz, 2009), thus it is not always clear when test score changes are meaningful in the short versus long term and whether those changes in test scores (whether gains or losses) are necessarily reflected in long-term educational or life outcomes (Brown et al., 2021). This is in parallel to the Flynn effect literature, where it is not always clear whether test score changes are due to genuine ability changes, noise, or specific aspects of the measures themselves (e.g., Rodgers, 1998).

From the perspective of cognitive ability researchers, at least, there are a number of empirically established findings that have accumulated over the past decades that would seem to greatly matter for those interested in pandemic loss or recovery to more deeply consider as a starting point for thinking about the importance of test score changes. First is the fact that the measurable constructs from ability and achievement tests are arguably more or less the same (Coleman & Cureton, 1954; Kaufman et al., 2012; Kelley, 1927) when considering construct(s) from the established structure of cognitive abilities (Carroll, 1993). Second, the research on the development of cognitive ability level and structure across the lifespan (Tucker-Drob, 2009) and the large literature showing the stability of general cognitive ability from infancy to adulthood (Breit et al., 2024; Gow et al., 2011; Gustavson et al., 2025) are also simply not cited or discussed in education. Third, the literature suggesting that abilities or cognitive aptitudes are current developed capacities that an individual brings to a specific problem-solving environment (Lohman, 2005; Snow, 1996; Subotnik et al., 2011) and that such developed abilities are important to problem solving environments such as school but are also can be enhanced or are a product of school is also ignored (Ceci, 1991; Lohman, 1993; Ritchie & Tucker-Drob, 2018). However, from the perspective of the field of education, empirical evidence may not necessarily be the main criterion motivating much of the research and policy debate (e.g., see Wai & Worrell, 2021, for one possible explanation for the lack of integration of evidence across the fields of intelligence and education).

Additionally, even though a large body of literature demonstrates a close association between ability and achievement (e.g., Deary et al., 2007; Roth et al., 2015), models of intelligence such as Carroll’s (1993) three-stratum theory and the Cattell-Horn-Carroll (CHC) model suggest subtle differences between them theoretically. CHC theory considers cognitive ability as a hierarchical structure, where broad abilities, such as fluid intelligence (*Gf*) and crystallized intelligence (*Gc*), including more narrow skills, nest under a general cognitive factor (*g*) (Carroll, 1993; Schneider & McGrew, 2012). While *Gf* includes inductive and deductive reasoning abilities, academic achievement scores primarily reflect crystallized knowledge acquired through formal instruction, which is drawn on related reasoning and problem-solving skills associated with *Gf*. Therefore, observed achievement scores should not be considered as direct measures of ability; achievement outcomes might be viewed as the transformation of cognitive ability mediated by particular educational conditions and learning opportunities. Moreover, skill formation theory conceptualizes that academic achievement is the outcome of a process of interaction between cognitive ability and educational inputs over time, in which cognitive ability does not translate directly into test performance (Cunha & Heckman, 2007). Within this approach, achievement reflects how effectively underlying cognitive abilities are transformed through instructions and learning opportunities. From this perspective, external shocks such as pandemic-related school closures and instructional disruptions may also weaken the relationship between achievement and ability.

Furthermore, the extent of disruptions to the ability-achievement association may not be uniform across students. Research on educational inequality and opportunity gaps suggests that students in different achievement distributions experience differential instruction quality and resource constraints (Reardon, 2011). Similarly, Kuhfeld et al. (2022) also showed variations in learning loss during COVID-19 depending on socio-economic conditions. Quantile regression is therefore theoretically an appropriate analytical approach in this context, as it allows for the investigation of distributional variations and changes in inequality that are not captured by OLS regression or mean-based analyses (Koenker & Hallock, 2001; Konstantopoulos et al., 2019). By demonstrating the ability-achievement relationship across the full achievement distribution, quantile regression may assess whether pandemic-related disruptions changed this association differently for lower, middle, and higher-achieving students (Konstantopoulos et al., 2019). Understanding these nuances, especially exploring ability-achievement links across quantiles, can also offer a policy lever, potentially distinguishing between changes in ability and changes in the ability-achievement relationship across achievement distributions, which may inform learning recovery strategies. Moreover, in the realm of the COVID-19-learning loss literature, almost no attention has been paid to the examination of the relationship between cognitive ability and observed academic achievement (largely because this is a topic that intelligence researchers focus on but is ignored by education researchers). Drawing such a distinction is important because declines in achievement test scores may indicate changes in skills, suggesting that policies based only on score drops may misdirect resources (Heckman et al., 2014).

Our study builds on the two frameworks described in this brief literature overview on pandemic learning losses and recovery from the education research and policy literature, and the longstanding research on how cognitive abilities are important measurable constructs in all cognitive tests. We do so by focusing on a unique dataset of two cohorts of students from a specific region of the state of Arkansas who took two different types of tests: an “ability” test and an “achievement” test, and use the method of quantile regression as a novel approach to assess any learning changes. Within this sample, our research questions are as follows:

Research Questions

RQ1: Did demographic gaps in cognitive ability widen, narrow, or shift after COVID-19?

RQ2: Did the relationship between cognitive ability and academic achievement change after COVID-19 across achievement levels?

## 2. Methods

### 2.1. Data and Sample 

The anonymized student-level assessment and demographic data used in this study were collected from the Arkansas Department of Education. With data from 15 school districts in Northwest Arkansas, we examined two cohorts of students, pre- and post-COVID-19 groups. For Cohort 1, the pre-COVID-19 Cohort (2018–19 school year), students sat for their achievement test (ACT Aspire) in 3rd grade and cognitive ability test (CogAT) in 4th grade in the 2019–20 school year. For Cohort 2, or the post-COVID-19 Cohort, students sat for an achievement test and an ability test in the 2021–22 school year during 4th grade. All the students in the sample across cohorts took both tests.

We used a total sample of 10,338 students, comprising 5277 in the pre-pandemic cohort and 5061 in the post-pandemic cohort. Table 1 reports summary statistics of their demographic characteristics. Across our samples, 53.41% of students were free and reduced price lunch (FRL) eligible, 49.36% were female, 23.70% received English Language Learner (ELL) services, 51.75% were white, 3.13% were Black, 34.71% were Hispanic, and 12.90% were identified as gifted and talented (G/T).

Student demographic data were collected from administrative records and measured concurrently with the assessment year. FRL was used as a proxy for economic disadvantage or lower socio-economic background, which is coded as a binary variable, taking one if a student is eligible for FRL and zero otherwise. Similarly, ELL is also defined based on the designation during the assessment and coded as a binary variable.

Additionally, the sample studied does not contain any missing data on CogAT or ACT Aspire outcomes. However, one school district lacked FRL information in the data due to privacy concerns, but we conducted robustness checks both excluding that district and excluding FRL from the models, and all results remained consistent.

### 2.2. Instruments

This study used scores from two tests: the ACT Aspire as an achievement test, and the CogAT as an ability test. The ACT Aspire is a vertically scaled achievement test that is used as a summative assessment of student achievement. It aims to measure grade-level and curriculum-aligned knowledge in English, reading, writing, math, and science (Williamson, 2019). The ACT Aspire includes batteries of achievement tests designed to measure student growth in a longitudinal assessment system for third through tenth grades (ACT Aspire, n.d.). For this study, we used the scale scores of math and English Language Arts (ELA) provided by the state and standardized them within grade and cohort.

The CogAT aims to measure verbal, quantitative, and nonverbal reasoning among K-12 students (Lohman & Lakin, 2010). These batteries assess basic reasoning processes that support academic learning. In this study, we used the Universal Scale Score (USS) or composite score. Although students completed the full CogAT battery, we only have the USS score data, which is a limitation of our analysis.

### 2.3. Analytical Approach

For exploring the cognitive ability gaps and trends of the achievement and ability test link, as guided by our research questions, we employed quantile regressions. The reason we chose this method is that quantile regressions, as opposed to ordinary least squares (OLS), which estimates only average effects, capture how cognitive ability and achievement differ across the entire distribution rather than only at the mean (Koenker & Hallock, 2001; Konstantopoulos et al., 2019). As education interventions often offer varied test score outcomes among low- and high-performing students (Konstantopoulos et al., 2019), examining quantiles is a necessary step to identify whether COVID-19-related changes disproportionately impacted certain parts of the distribution. For RQ1, we used the following quantile regression model:

$$Q_{\tau }(Y_{i})=\alpha_{\tau }+\beta_{1\tau }X_{i}+\beta_{2\tau }{COVID}_{i}+\beta_{3\tau }(X_{i}\times {\mathrm{COVID}}_{i})+\epsilon_{i\tau }$$

where, $Q_{\tau }$ refers to the quantiles—0.10, 0.25, 0.50, 0.75, and 0.90. $Y_{i}$ indicates our outcome variable, the standardized cognitive ability test (CogAT) score, while $X_{i}$ includes the demographic and programmatic variables, such as students’ socioeconomic status and race/ethnicity. ${COVID}_{i}$ is a binary variable, it takes 0 for pre-pandemic Cohort 1, and takes 1 for post-pandemic Cohort 2. $\beta_{3\tau }$ represents the interaction terms between ${COVID}_{i}$ and demographic variables, providing the coefficients of interest.

For RQ2, we used the following quantile regression model:

$$Q_{\tau }(Y_{i})=\alpha_{\tau }+\beta_{1\tau }{\mathrm{CogAT}}_{i}+\beta_{2\tau }{\mathrm{COVID}}_{i}+\beta_{3\tau }({\mathrm{CogAT}}_{i}\times {\mathrm{COVID}}_{i})+\gamma_{\tau }X_{i}+\epsilon_{i\tau }$$

Here, $Y_{i}$ is our outcome variable, which includes achievement test scores (ACT Aspire). The coefficients of $\beta_{3\tau }$ are of interest, because this interaction term between ${COVID}_{i}$ and ability test scores can demonstrate the associations between achievement and ability across the pre-and post-pandemic cohorts.

## 3. Results

RQ1: Did demographic gaps in cognitive ability widen, narrow, or shift after COVID-19?

For this research question, we estimated the quantile regression of standardized CogAT scores across the cognitive distribution. The results consistently reveal that the cognitive ability gaps did not widen after the pandemic, instead they stayed stable or narrowed moderately across groups. For example, before COVID-19, students with economically disadvantaged backgrounds scored 0.33 SD lower at the 10th, 0.43 SD lower at the 25th, 0.43 SD lower at the 50th, 0.54 SD lower at the 75th, and 0.65 SD lower at the 90th percentiles, compared to their economically advantaged peers (*p* < 0.001). After the pandemic, the interaction term between the post-COVID-19 cohort and economic disadvantage produced positive coefficients, such as +0.12 SD (τ = 0.10), +0.28 SD (τ = 0.25), +0.33 SD (τ = 0.50), +0.39 SD (τ = 0.75), and +0.50 SD (τ = 0.90), suggesting substantial reduction of gaps across the entire cognitive distribution (see Table 2). This implies that the cognitive ability gap between students from disadvantaged backgrounds and their counterparts did not widen after the pandemic, rather it became narrowed to some extent.

Similarly, gaps for ELL students were large before COVID-19 but narrowed afterwards. before COVID-19, ELL students scored 0.76 SD lower at the 10th, 0.92 SD lower at 25th, 0.81 SD lower at 50th, 0.81 SD lower at the 75th and 1.03 SD lower at 90th percentiles, compared to their non-ELL peers (*p* < 0.001). After COVID-19, the interaction term between post-COVID-19 and ELL status produced positive coefficients, such as +0.35 SD (τ = 0.10), +0.46 SD (τ = 0.25), +0.32 SD (τ = 0.50), +0.35 SD (τ = 0.75), and +0.62 SD (τ = 0.90), indicating a noticeable reduction of gaps across all cognitive distributions.

Gender gaps in cognitive ability remained relatively stable. Before COVID-19, at the 10th and 25th percentiles, female students were 0.16 and 0.11 SD above male students, but for the 75th and 90th percentiles were 0.11 SD and 0.27 SD lower than male students (*p* < 0.001). After COVID-19, the results did not reveal any statistically significant (at the 99% or 95% level) gender differences, suggesting no noticeable widening or narrowing.

Racial/ethnic gaps also did not widen. Pre-COVID-19 gaps between Black and non-Black students ranged from approximately 0.38 SD to 0.70 SD (*p* < 0.05 to *p* < 0.001), depending on quantile; the post-COVID-19 interactions were generally positive or lacked statistical significance at the 99% or 95% level. Hispanic students also showed no signs of widening cognitive gaps after COVID-19. Prior to the pandemic, Hispanic students showed a slight advantage compared to non-Hispanic students at the lower end of the CogAT distribution; however, post-COVID-19 results demonstrate some slight differences across the 10th and 25th percentiles. Asian students appear to have shown a slight advantage before COVID-19 compared to non-Asian students, but post-COVID-19 results show no statistically significant gaps. White students demonstrated a different trend than other racial and ethnic groups. They had no statistical difference with non-White students before COVID-19, but after the pandemic, they gained 0.26 SD to 0.46 SD (*p* < 0.05 to *p* < 0.001) at the middle and upper parts of the CogAT distribution.

Although Asian and Hispanic students experienced small declines relative to their counterparts at certain points in the ability distribution, and White students showed modest gains at the middle and upper quantiles, these shifts were limited in size and did not indicate any broader trend toward increased cognitive inequality.

Overall, the results suggest a stable cognitive distribution across cohorts between the pre- and post-COVID-19 periods, with small differences. The magnitude of such differences between pre- and post-pandemic cohorts is estimated well below one-tenth of an SD across distributions. From a practical perspective, these effect sizes suggest no meaningful widening of cognitive ability gaps after COVID-19. These findings are robust across alternative specifications. Estimated demographic gaps and coefficients with or without control variables and district-level fixed effects remain consistent. Also, the consistency of the findings across the quantiles supports the robustness of these results.

RQ2: Did the relationship between cognitive ability and academic achievement change after COVID-19 across achievement levels?

To examine the relationship trends between cognitive ability (CogAT) and academic achievement (ACT Aspire) test scores before and after the pandemic, we conducted another set of quantile regressions. Results demonstrated that the association between cognitive ability and achievement was strong before COVID-19, but the magnitude of this relationship weakened significantly after the pandemic. To be specific, before COVID-19, a one SD increase in cognitive ability score was associated with an increase of 0.68 SD at the 10th, 0.65 SD at the 25th, 0.61 SD at the median, 0.56 SD at the 75th, and 0.49 SD at the 90th percentiles in achievement test scores, holding all else equal (*p* < 0.001). However, after the pandemic, these associations became weaker, regardless of whether a student was low-achieving or high-achieving. To calculate the post-pandemic trends, we calculated the effects of the interaction terms using the following formula:

βpost = βpre + interaction

The results revealed (see Table 3) that after the pandemic, a one SD increase in cognitive ability scores was associated with an increase of 0.31 SD at the 10th, 0.36 SD at the 25th, 0.43 SD at the median, 0.46 SD at the 75th, and 0.42 SD at the 90th percentiles in achievement test scores, holding all else equal (*p* < 0.001). This suggests a clear trend of a weaker ability-achievement link across all distributions after COVID-19, with the largest gap among lower-achieving students.

Overall, because both CogAT and ACT Aspire scores are standardized, the coefficients can be interpreted as effect sizes. Therefore, in summary, before COVID-19, a one SD increase in cognitive ability scores predicted 0.49–0.68 SD higher scores on the achievement test, depending on quantile. However, after the pandemic, these metrics dropped to 0.31–0.46 SD. One noticeable pattern is that the relationship was weakest among the lowest-performing students. In other words, the transformation mechanism of cognitive ability into academic achievement became significantly weaker after COVID-19, and the process was worse among the lower quantile students. Practically, these results imply that post-pandemic achievement scores became a less reliable signal of lower quantile students’ underlying developed cognitive ability.

Given the consistency of the coefficients and standard errors, these findings appear to be robust. However, for reassurance, we ran some additional Pearson correlations to check their robustness. The results showed that the correlation between cognitive ability and achievement was 0.72 before COVID-19, but it reduced to 0.46 after COVID-19, suggesting the robustness of these results.

## 4. Discussion

Overall, we found that demographic gaps in cognitive ability did not change from before to after COVID-19 and that the relationship between the ability and achievement test changed, mostly at the lower quantiles, across pre-and post-pandemic. This aligns well with the prior literature supporting the stability of cognitive abilities (e.g., Breit et al., 2024; Gow et al., 2011; Gustavson et al., 2025). It also makes sense, given the relationship at the top quantiles, when building on the education and policy findings from Arkansas, which showed that at least two of the districts showed generally strong recovery after the pandemic on both math and reading (Fahle et al., 2024).

**Figure A1:**
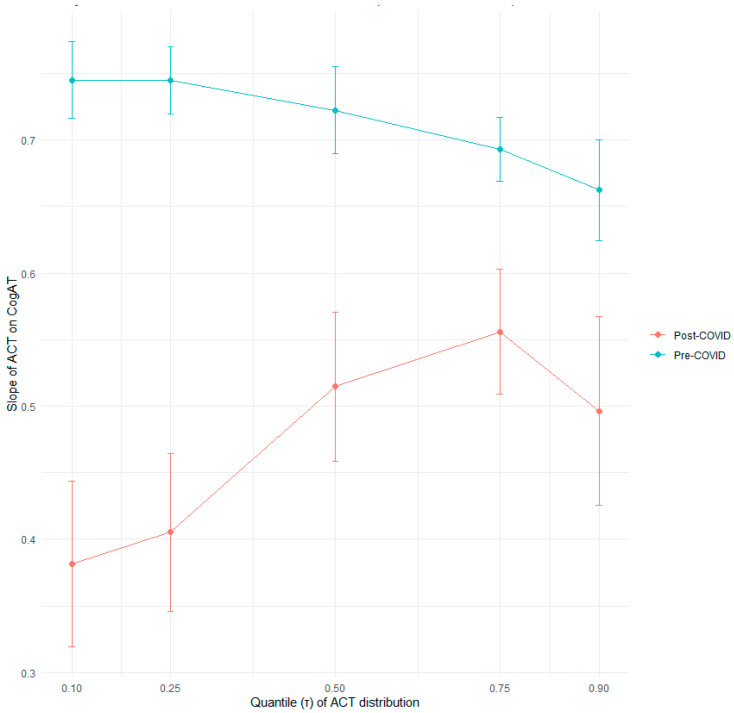
Ability-achievement link across distributions (Pre- vs. post-COVID-19).

Broadly, the association between ability and achievement test scores appeared statistically significant across both cohorts, findings that align with a large body of research that establishes a strong ability-achievement link (e.g., Deary et al., 2007; Roth et al., 2015). However, our findings also showed a weaker post-pandemic ability-achievement link (See Figure A1 in Appendix A), or in other words, the potential disruption in educational mechanisms that normally transform ability into observed achievement performance. This resonates with the recent evidence demonstrating that the downstream returns to education are likely to be overstated in the literature, implying fragility in the translation from schooling to outcomes rather than changes in underlying inputs (Clark & Abildgaard Nielsen, 2026). A skill-formation perspective provides a theoretical framework for understanding this fragility. According to Cunha and Heckman (2007), the full expression of cognitive ability into achievement depends on instructional input and opportunities to practice (see also Subotnik et al., 2011). Accordingly, pandemic-related disruptions, such as reduced instructional time, school closures, and uneven access to quality learning environments, may have disproportionately affected students at the lower end of the achievement distribution, even if their underlying developed cognitive ability remained relatively unchanged. Prior research demonstrates academic achievement differences often reflect unequal access to instructional resources and learning opportunities (Reardon, 2011), and achievement declines happened more noticeably among the students from disadvantaged backgrounds during COVID-19 (Kuhfeld et al., 2022), suggesting resource constraints, i.e., limited access to educational resources and opportunities, as a plausible explanation for the weakened ability-achievement link among lower-quantile students in the post-pandemic period that we uncovered.

These findings suggest that post-pandemic recovery initiatives should not be driven by a uniform remediation strategy solely based on average observed achievement declines. First, the evidence that cognitive ability remained stable while its translation into achievement became weak after COVID-19 suggests a focus on strengthening learning supports that may facilitate the transformation of ability to achievement among lower quantile students. These efforts may include structured instructional scaffolding, learning strategy training, or consistent academic feedback. Additional supports that address non-cognitive barriers, such as learning motivation and academic self-efficacy with formal school routines, may also be useful.

Second, the varied magnitude of the ability-achievement link between the lower and upper quantiles across the pre- and post-COVID-19 periods suggests differentiated remediation strategies that might more fruitfully translate students’ cognitive ability into achievement. Because the ability distributions remained stable, generalized remediation strategies may not necessarily help repair the translation from ability to achievement. Perhaps including targeted support for lower-performing students with intact cognitive ability might be useful. At the same time, it may be that the pandemic did not have as much of an impact as many in the education community have claimed.

Additionally, we used the novel approach of using quantile regressions to examine changes across the ability range, which provides stronger evidence than studies that simply look at the average overall means of test score changes without adequately accounting for ability heterogeneity. Much of the literature on COVID-19-related learning loss has focused on average declines in test scores (e.g., Adelman, 2025; Cruz et al., 2025; Fahle et al., 2024), which can limit the meaningful understanding of heterogeneity across students. In contrast, the key contribution of this study is the demonstration of the achievement score changes in post-COVID-19 times across lower, middle, and higher distributions, providing a more nuanced picture of how educational disruptions may have affected achievement distributions (Koenker & Hallock, 2001).

## 5. Limitations and Future Directions

A clear limitation of this study is that we were only able to study one achievement and one cognitive ability measure in a sample of 15 districts in Arkansas, which are not representative of the state as a whole. Though there were small changes for different subgroups in our study, these are relatively minor in the overall pattern of findings. Of course, future research might be conducted by following up these studied cohorts into the future by examining their long-term outcomes.

Additionally, as we did not have the 4th-grade achievement data of the pre-COVID cohort, we used their 3rd-grade achievement data. However, since CogAT scores in both cohorts were from the same grade level (Grade 4), cross-grade comparisons were not an issue for CogAT. For ACT Aspire, we did not use raw Grade 3 and Grade 4 scale scores directly. All analyses used scores standardized within grade and cohort. Also, as an additional check, we recomputed the correlations using the Grade 3 achievement score in both cohorts, and the results were substantively unchanged.

## Figures and Tables

**Table 1:** Student demographic characteristics.

Variables	Overall		Cohort 1		Cohort 2	
	*N*	*%*	*N*	*%*	*N*	*%*
*Gender*						
Female	5103	49.36	2604	49.35	2499	49.38
Male	5235	50.64	2673	50.65	2562	50.62
*Race and Ethnicity*						
Asian	204	1.97	100	1.90	104	2.05
Black	323	3.13	131	2.48	192	3.79
Hispanic	3587	34.71	1895	35.94	1692	33.43
White	5347	51.75	2645	50.17	2702	53.39
Other race	872	8.44	501	9.50	371	7.33
*Educational Characteristics*						
ELL	2450	23.70	1385	26.25	1065	21.04
FRL	4718	53.41	2039	54.06	2679	52.93
G/T	1334	12.90	694	13.15	640	12.65

*Note.* Data for FRL status in Cohort 1 were not provided by one school district due to privacy concerns.

**Table 2:** Quantile regression of cognitive ability on demographics and COVID-19.

Variables	(1)	(2)	(3)	(4)	(5)
	τ = 0.10	τ = 0.25	τ = 0.50	τ = 0.75	τ = 0.90
FRL	−0.32 ***	−0.43 ***	−0.43 ***	−0.54 ***	−0.65 ***
	(0.06)	(0.04)	(0.04)	(0.05)	(0.06)
Post-COVID 19	−0.18	−0.37 ***	−0.37 **	−0.53 ***	−0.68 **
	(0.16)	(0.10)	(0.07)	(0.11)	(0.23)
FRL × Post-COVID19	0.12	0.28 ***	0.33 ***	0.39 ***	0.50 ***
	(0.09)	(0.06)	(0.07)	(0.07)	(0.10)
Female	0.16 ***	0.11 **	−0.00	−0.11 *	−0.27 ***
	(0.06)	(0.05)	(0.03)	(0.05)	(0.06)
Female × Post-COVID19	−0.06	−0.01	−0.03	0.06	0.12
	(0.09)	(0.05)	(0.05)	(0.06)	(0.09)
ELL	−0.76 ***	−0.92 ***	−0.81 ***	−0.81 ***	−1.03 ***
	(0.07)	(0.06)	(0.04)	(0.06)	(0.10)
ELL × Post-COVID19	0.34 **	0.46 ***	0.32 ***	0.35 ***	0.62 ***
	(0.09)	(0.08)	(0.06)	(0.08)	(0.17)
White	0.00	−0.00	−0.08	−0.16	−0.21
	(0.21)	(0.17)	(0.11)	(0.15)	(0.30)
White × Post-COVID19	0.10	0.21	0.26 *	0.41 ***	0.46 *
	(0.15)	(0.14)	(0.08)	(0.12)	(0.21)
Black	−0.21	−0.38	−0.51 **	−0.70 ***	−0.59
	(0.27)	(0.17)	(0.16)	(0.25)	(0.32)
Black × Post-COVID19	0.06	0.33	0.36 *	0.59 **	0.43
	(0.22)	(0.21)	(0.16)	(0.20)	(0.31)
Hispanic	0.38	0.38	0.14	0.06	−0.04
	(0.20)	(0.16)	(0.12)	(0.15)	(0.26)
Hispanic × Post-COVID19	−0.33 *	−0.27 *	−0.19	−0.17	−0.27
	(0.15)	(0.09)	(0.07)	(0.12)	(0.16)
Asian	0.27	0.48 *	0.30	0.50 **	0.39
	(0.24)	(0.20)	(0.17)	(0.23)	(0.42)
Asian × Post-COVID19	−0.33	−0.02	−0.02	−0.08	0.18
	(0.29)	(0.27)	(0.14)	(0.27)	(0.37)
Other races	−0.05	0.05	−0.08	−0.16	−0.21
	(0.17)	(0.13)	(0.10)	(0.12)	(0.21)
Constant	−0.92 ***	−0.27	0.46 **	1.18 ***	2.05 ***
	(0.22)	(0.15)	(0.12)	(0.13)	(0.29)
N	8828	8828	8828	8828	8828
*R* ^2^	0.05	0.07	0.08	0.09	0.10

*Note.* Standard errors in parentheses. One school district did not provide FRL data for the pre-COVID-19 Cohort, which reduced the number of observations in the regression. * *p* < 0.05, ** *p* < 0.01, *** *p* < 0.001.

**Table 3:** Relationship between cognitive ability and academic achievement after COVID-19.

Variables	(1)	(2)	(3)	(4)	(5)
	τ = 0.10	τ = 0.25	τ = 0.50	τ = 0.75	τ = 0.90
CogAT Standardized Score	0.68 ***	0.65 ***	0.61 ***	0.56 ***	0.49 ***
	(0.02)	(0.02)	(0.02)	(0.02)	(0.02)
Post-COVID 19	−0.17 ***	−0.09 ***	0.02	0.06 ***	0.16 ***
	(0.02)	(0.03)	(0.03)	(0.02)	(0.02)
CogAT × Post-COVID 19	−0.37 ***	−0.29 ***	−0.18 ***	−0.10 ***	−0.07 ***
	(0.02)	(0.03)	(0.03)	(0.03)	(0.02)
FRL	−0.22 ***	−0.28 ***	−0.26 ***	−0.26 ***	−0.33 ***
	(0.04)	(0.02)	(0.02)	(0.03)	(0.03)
Female	0.09 **	0.04	0.08 ***	0.09 ***	0.11 ***
	(0.03)	(0.02)	(0.02)	(0.01)	(0.03)
ELL	−0.48 ***	−0.46 ***	−0.48 ***	−0.48 ***	−0.49 ***
	(0.03)	(0.03)	(0.03)	(0.02)	(0.04)
Asian	0.22 **	0.14 **	0.15 *	0.04	0.04
	(0.08)	(0.06)	(0.08)	(0.10)	(0.08)
Black	−0.12	−0.17 ***	−0.02	−0.09 *	−0.10
	(0.13)	(0.06)	(0.09)	(0.04)	(0.08)
Hispanic	0.22 ***	0.20 ***	0.17 ***	0.09 **	0.03
	(0.03)	(0.03)	(0.03)	(0.03)	(0.05)
Other race	0.17 **	0.11 **	0.03	−0.12 ***	−0.20 ***
	(0.07)	(0.04)	(0.04)	(0.04)	(0.04)
Constant	−0.79 ***	−0.28 ***	0.18 ***	0.70 ***	1.13 ***
	(0.05)	(0.03)	(0.02)	(0.02)	(0.02)
N	8828	8828	8828	8828	8828
*R* ^2^	0.21	0.22	0.23	0.25	0.24

*Note.* Standard errors in parentheses. Comparison group for the races (Asian, Black, Hispanic, and other races) was White. * *p* < 0.05, ** *p* < 0.01, *** *p* < 0.001.

## Data Availability

Due to privacy restrictions, data are not publicly available.
